# Towards BCI-actuated smart wheelchair system

**DOI:** 10.1186/s12938-018-0545-x

**Published:** 2018-08-20

**Authors:** Jingsheng Tang, Yadong Liu, Dewen Hu, ZongTan Zhou

**Affiliations:** College of Artificial Intelligence, National University of Defense Technology, Deya Road, Changsha, 410000 People’s Republic of China

## Abstract

**Background:**

Electroencephalogram-based brain–computer interfaces (BCIs) represent novel human machine interactive technology that allows people to communicate and interact with the external world without relying on their peripheral muscles and nervous system. Among BCI systems, brain-actuated wheelchairs are promising systems for the rehabilitation of severely motor disabled individuals who are unable to control a wheelchair by conventional interfaces. Previous related studies realized the easy use of brain-actuated wheelchairs that enable people to navigate the wheelchair through simple commands; however, these systems rely on offline calibration of the environment. Other systems do not rely on any prior knowledge; however, the control of the system is time consuming. In this paper, we have proposed an improved mobile platform structure equipped with an omnidirectional wheelchair, a lightweight robotic arm, a target recognition module and an auto-control module. Based on the you only look once (YOLO) algorithm, our system can, in real time, recognize and locate the targets in the environment, and the users confirm one target through a P300-based BCI. An expert system plans a proper solution for a specific target; for example, the planned solution for a door is opening the door and then passing through it, and the auto-control system then jointly controls the wheelchair and robotic arm to complete the operation. During the task execution, the target is also tracked by using an image tracking technique. Thus, we have formed an easy-to-use system that can provide accurate services to satisfy user requirements, and this system can accommodate different environments.

**Results:**

To validate and evaluate our system, an experiment simulating the daily application was performed. The tasks included the user driving the system closer to a walking man and having a conversation with him; going to another room through a door; and picking up a bottle of water on the desk and drinking water. Three patients (cerebral infarction; spinal injury; and stroke) and four healthy subjects participated in the test and all completed the tasks.

**Conclusion:**

This article presents a brain-actuated smart wheelchair system. The system is intelligent in that it provides efficient and considerate services for users. To test the system, three patients and four healthy subjects were recruited to participate in a test. The results demonstrate that the system works smartly and efficiently; with this system, users only need to issue small commands to get considerate services. This system is of significance for accelerating the application of BCIs in the practical environment, especially for patients who will use a BCI for rehabilitation applications.

## Background

An electroencephalogram (EEG)-based brain–computer interface (BCI) is a novel human–machine interactive technology that allows people to communicate and interact with the external world without relying on their peripheral muscles and nervous system [1]. Among BCI systems, brain-actuated wheelchairs are promising systems for the rehabilitation of severely motor disabled individuals who are unable to control the wheelchair by conventional interfaces. In recent years, extensive progress has been made on brain-actuated wheelchairs.

Early brain-actuated wheelchair systems were straightforward and were implemented by applying a BCI to a wheelchair. The BCI system acts as an alternative controller, such as a joystick, that directly controls the wheelchair. For example, in [2], the user controls the directions of the wheelchair through mental tasks. Due to more extensive research, many more wheelchair functions, such as start/stop and the acceleration/deceleration can now be achieved by different kinds of BCIs, e.g., P300 BCIs [3, 4], steady-state visual evoked potential (SSVEP) BCIs [5, 6], motor imagery (MI)-based BCIs [7, 8], and even hybrid BCIs [9–12].

With the growing number of studies on this topic, researchers have introduced the shared control [13] framework into BCI-actuated systems to improve the security and performance of the BCI systems. In such a system, the device is equipped with automation control technology to build a semiautonomous system that works in cooperation with humans. Researchers equipped the wheelchair with sensors such as radar, lasers and vision camera to capture the environmental context, to enhance control to avoid obstacles or to correct an improper command issued by the BCI [14–19]. For example, Millar et al. presented their system, which is based on comprehensively analysing data from the human brain and the environmental data captured by a laser range finder (LRF), to build a context filter to filter incorrect BCI commands and ensure security during navigation. They also developed another system to smooth the moving trajectory based on the sensor’s data and human intent. From the perspective of human–machine interactions, the automation control module in these systems works via a low level shared control framework to correct or optimize the driving commands; however, the user still directly controls the wheelchair.

The basic function of a wheelchair is to transport a person from place A to place B, and the details of wheelchair control are not necessary for users to know. An intuitive, easy-to-use system for users, especially patients, is highly important. Some researchers have proposed systems that function in such a way. In the work of Iturrate et al. [3], which was based on virtual reality technology, the scenario of the environment is reconstructed and displayed on a screen, and a predefined N × M polar grid is used to define a set of destinations (destinations outside the accessible area are automatically eliminated). Users select a destination through a P300-based BCI, and as long as the destination is confirmed, the system automatically navigates to the destination. In contrast to Iturrate’s system’s [3] real time reconstructing scenario, there are several systems that predetermine the destinations of the target, with the users steering the wheelchair by choosing one goal through the BCI. For example, Rebsamen et al. [20] proposed a system that works in familiar environments with target locations such as bed, the television, a desk, all being predetermined. The user chooses one target by a P300-based BCI, the path to the goal is generated by the computer, and the wheelchair can auto navigate to the goal. Zhang et al. [21] proposed a similar system, in which they mounted two webcams on the wall to predetermine the locations of the targets, and the wheelchair was equipped with a laser so that the system could dynamically plan a safe trajectory to an assigned destination. Users also steer the wheelchair by indicating the intended goal through the BCI. Another system proposed by Lopes et al. [22] also predetermines the waypoints and goals offline and uses a P300-based BCI to provide five steering commands: go forward, turn left 45°, turn left 90°, turn right 45° and turn right 90°. The system determined the most likely destination according to the current BCI command and the distribution of the targets. The advantages and disadvantages of these systems are obvious: they represent smart mobile solutions. The systems with a straightforward solution to predetermining the goals in the environment can reliably work in familiar environments, however, any changes in the environment require the system to recalibrate the goals, and these systems are unable to deal with dynamic goals such as people. Iturrate’s system [3] avoids this problem, because their system does not rely on any prior experience; all the necessary information is captured online. However, the destinations are defined by a grid, which means the marked destination does not represent the real target; getting to one destination requires multiple destination selections and validations to gradually get closer to the real target, therefore this system is not very intuitive and is time consuming.

Our team is also engaged in building intuitive and efficient mobility solutions for users. We have employed target recognition technology and auto navigation technology to build a target driven and dynamic system. Specifically, the target recognition module recognizes and locates the target in the environment online, the user confirms one target by selecting this target directly through a BCI system, and the auto navigation module steers the wheelchair to the assigned destination. Thus, this system can deal with a dynamic environment, and the process of approaching a target is straightforward. Additionally, our work goes further in that we consider that a specific purpose commonly accompanies navigation tasks; for example, moving closer to a desk is often for the purpose of picking something up. Thus, we have equipped the wheelchair with a lightweight robotic arm as an additional actuator. Based on the target recognition result, we plan a proper solution by comprehensively considering the properties of the target, the context of the current condition and other factors for a target. For example, we would plan a solution for a bottle of water as the user approaches it by picking up it and feeding the user. Accordingly, the mobility of the system is enhanced (for example, opening the door before entering a room), and the application of the system is broadened (i.e., go to somewhere to do something). To validate and evaluate our system, an experiment simulating daily application was performed. The tasks included the user driving the system closer to a walking man to have a conversation with him; going to another room through a door; and picking up a bottle of water on the desk and drinking water. Three patients (cerebral infarction; spinal injury; and stroke) and four healthy subjects participated in the test and all completed the tasks.

## Methods

### Smart wheelchair system

**Fig. 1:**
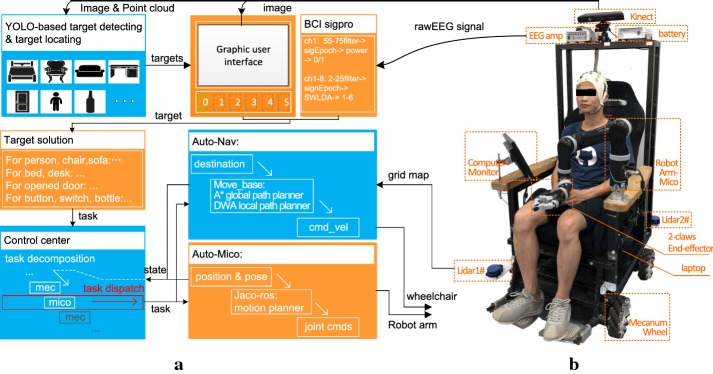
The structure of our system. **a** System modules of our system. **b** Photograph of our system

Figure 1 illustrates the architecture of the system. The smart wheelchair system was designed using artificial intelligence technology to enable the system to adapt to dynamic environments and to intelligently complete operations. With a BCI system, users operate the wheelchair in a simple and intuitive manner. In this section, the four parts of the smart wheelchair system are introduced. These parts are: the hardware and software structure; target detection and localization; the target solution; and the wheelchair and robotic arm control.

#### Hardware and software structure

##### Hardware structure

In this system, a flexible wheelchair was constructed by introducing an omnidirectional chassis. This chassis is based on the mecanum wheel [23], which allows for the wheelchair to travel in any direction and to rotate with zero radius; thus, the wheelchair can better accommodate navigation in complicated environments, for example, small spaces or a domestic house with a lot of furniture. Considering that the users of the system may be severely disabled people, we also introduced a lightweight five degrees of freedom (DOF) robotic arm (Mico, Kinova, Canada) equipped with a two claw end effector into the system and mounted it on the left armrest. Thus, the robotic arm can provide assistance for navigation tasks (for example by opening the door before entering a room.) to effectively broaden the activity range of the wheelchair without requiring help from others and can aid in completion of navigation-subsequent tasks (for example picking up a bottle of water) since, as we have stated, navigation tasks commonly have a certain purpose. In addition, several other components are equipped as follows:

- *Kinect camera* A Kinect camera is mounted on the back support of the wheelchair at a height of approximately 1.5 m with a 0.37° depression angle to capture the RGB and depth streams at the front of the wheelchair over a sector area covering an approximately 57° visual angle. The camera provides the system with 640 × 480 RGB images at 20 frames per second (FPS), and by merging the depth stream, the 3D point cloud of the scene is obtained. In our test runs, the location error of the 3D point cloud was approximately 1 cm within a 2 m area. This level of accuracy enables our system to operate on objects no less than 2 cm in size, such as bottles and ordinary electric buttons. During the experiment, the 3D points of the wheelchair body and the points below the height of 10 cm from the floor were eliminated to accelerate the calculations.
- *Low cost lidars* On the front right corner and back left corner of the wheelchair, two single line low cost lidars are mounted at a height of 35 cm above the floor. Each lidar is able to measure objects in the environment with a 1° angle resolution in a 0.1–8 m range at 5 Hz. The calibration programme is performed to calculate the transforming matrix between the two coordinate systems. Thus, with this transforming matrix, the data from these two lidars are fused, and after eliminating the data in the range of the wheelchair itself, the surrounding environment’s measurement is obtained.
- *Other devices* In addition to the Kinect camera and the lidars, a USB camera is mounted on the back support of the wheelchair to capture the backward scene of the environment. A 12 in computer monitor is mounted on the right armrest of the wheelchair to display the stimulation interface of the BCI. A laptop with an independent graphics processing unit (GPU) to support the deep learning programming framework is equipped as the host computer.

##### Software structure

As described above, this smart wheelchair is composed of sensors, devices and corresponding computational modules. These modules communicate and cooperate with each other to complete tasks. To ensure the system works efficiently and can be easily managed, a good software structure is important. A robot operating system (ROS [24]) was employed to construct our system, since an ROS is a widely used programming tool for robot applications. This tool realizes hardware abstractions for common devices and sensors as well as many mature algorithms for robot control. One ROS application is split into independent nodes that are running in independent processes, and the nodes communicate with each other via a standard method through standard messages. Therefore, such applications are developer friendly and can be efficiently managed.

**Fig. 2:**
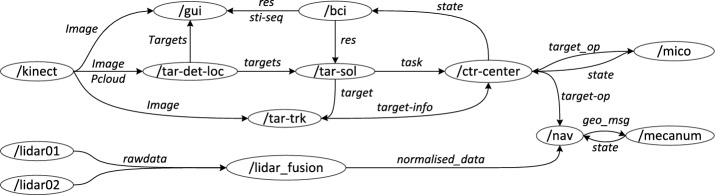
Node graph of our system’s software structure

The node graph of our system is illustrated in Fig. 2. There are thirteen main nodes: “/lidar01”, “/lidar02”, “/lidar_fusion”, “/mecanum”, “/nav”, “/mico”, “/Kinect”, “/tar-det-loc”, “/tar-sol”, “/gui”, “/bci”, “/ctr-center” and “/tar-trk”. The system runs at 10 Hz, and all the nodes communicate with each other through the ROS topic. The /lidar_fusion node subscribes the message of /lidar01 and /lidar02 to normalize their coordination system, fuses the measured data, eliminates data in the range of the wheelchair itself, and finally publishes the fused message. The /Kinect node captures the RGB and depth data of the environment. The /tar-det-loc node recognizes and locates the target based on the image and 3D point cloud from the Kinect. The /tar-sol node prepares solutions for the targets and publishes this information to the /gui. The graphical user interface (GUI) displays the image from the /Kinect and detected targets from the /tar-det-loc. The /bci node deals with the online EEG data and estimates the target selected by the user. The /tar-sol node subscribes the result of the BCI system and publishes the target position and corresponding solutions to the /ctr-center node. The/ctr-center node decomposes the tasks as control sequences such as mecanum move**->robot arm act**->mecanum move**. The control sequences are sent to the mecanum or robotic arm. At each control frame, the mecanum or robotic arm will report whether the current command is completed, and as long as one command sequence is completed, the next control sequence is to be executed. In particular, the image tracking node /tar-trk will update the target information for the system during task execution.

#### Target detection and location

To allow the system to intelligently serve the user in dynamic environments, we employed a target detection algorithm to recognize targets in the environment in real time. The RGB stream from the Kinect is used as the source image. The deep learning based target detection method, which has been rapidly developed in recent years, was used in this research, since this method has excellent performance in image processing compared with traditional computer vision methods. Specifically, in using the system in real time applications, the YOLOv2 [25], which exhibits high speed target detection, is employed in this system. Using our laptop, this method is able to achieve 15 FPS with the 640 × 480 RGB image, which is sufficiently fast for our system’s application. To accommodate our application, an image training database was built based on a sample of images (“chair”, “bed”, “sofa”, “person”, “cup” and “bottle”) from the Common Objects in Context dataset (COCO) [26] and images acquired by ourselves (“opened door”, “closed door”, “desk” and “electric switch”). With the pre-trained neural network published on YOLOv2’s official site, the programme was trained on this reorganized database, and consequently our system is able to recognize ten classes of objects that are commonly found in a domestic environment.

After target detection, the bounding box of the target is confirmed. To eliminate non-useful points, a smaller bounding box that is 60% of the original in size is used to extract the 3D points. The centre of these 3D points is calculated as the estimation of the target position. The orientation of the target is also estimated, because the orientation of the target significantly affects human interaction with that target. For example, the comfortable orientation for two people to communicate is face to face. To estimate the orientation of the detected object, we first assumed that the object was vertically positioned with respect to the floor, i.e., we considered only the orientation in the horizontal plane or the xoy plane in the coordination system of our system. We project the points of the object to the xoy plane and then find the principal orientation vector *v* by principal component analysis (PCA). Additionally, the vector *f* pointing from the wheelchair to the target centre is calculated. The angle between the *v* and *f* vectors and the angle between *vn* (i.e., the orthogonal vector of *v*) and *f* are tested, and the *v* or *vn* vector with the smaller angle to *f* is confirmed as the orientation of the target. Thus, the target’s position and orientation are confirmed.

However, during navigation, the relative position between the target and the system will change. Although this change can be estimated by recording the wheelchair’s movement, location error will be introduced, and this error is unacceptable for robotic arm operation (e.g., to pick up a bottle, the location error should be limited to 2 centimetres). Another consideration is that this system is designed to accommodate dynamic objects; therefore, the target object’s movement should also be taken into consideration. Therefore, during system operation, once the target is confirmed by the user, the target is tracked with image tracking technology [the kernelized correlation filter (KCF) [27] method is used], and the location and orientation are updated with each newly updated target bounding box. Thus, the system maintains precise positioning of the target.

#### Target solution

In this system, the following ten classes of targets can be recognized: “chair”, “opened door”, “closed door”, “bed”, “sofa”, “desk”, “person”, “bottle”, “electric switch”, and “cup”. Through the BCI system, the user can select one class of target. However, as discussed, the navigation tasks are commonly accompanied by special aims. Therefore, our system does not seek to just “transport one from place A to place B”; rather, it seeks to further predict the user’s intent to provide proper service for him and satisfy the user’s real demand. In our hypothesis, the attributes of the target, the context, and the habits and historical behaviour of the user can all be used as factors to infer the user’s intention and then provide him with the best service. As a preliminary attempt, we provide solutions for each target based on the attributes of the target. The ten targets are classified into four groups, and the corresponding solutions were defined as follows:

- For “person”, “chair” and “sofa”, we assume that the aim of the user selecting these targets is to have a conversation with a person or a person seated on a chair or sofa. Therefore, the solution is defined as stopping at a distance of 80 cm from the target (a comfortable distance for communication) and facing the target.
- For “bed”, “closed door” and “desk”, the solution is defined as reaching the target at a distance of 20 cm and facing the target, because there may be a subsequent operations that can be performed on this target.
- For “opened door”, the solution is defined as reaching the target and then passing through it.
- For “electric switch”, “bottle” and “cup”, the solution is defined as reaching the target and then pressing it or picking it up. Specifically, the optimal workspace of the robotic arm is pre-calibrated, and therefore, the operation is defined as first driving the wheelchair until the target enters the workspace of the robotic arm and then manipulating the robotic arm to the target. For a switch, the operation is to press it, and for a cup or bottle, the operation is to pick it up and translocate it to the user’s mouth.

#### Wheelchair and robotic arm control

The autonomous navigation system and the motion planning system were designed for wheelchair control and robotic arm control, respectively. The ROS package “move base” was used to build the navigation system. This package provides complete solutions for various types of robot navigation. For a given destination, it plans a global path in the initial state, and during navigation, the local planner plans the optimal path according to the real-time map to decide the proper velocity and orientation of the wheelchair at each control step. With this package, only a few parameters need to be set, such as the maximum/minimum velocities in the x/y directions (the maximum and minimum velocities are set to 0.4 and 0.1 m/s, respectively), the type of robot (which corresponds to the “holonomic robot” parameter; in this study, this parameter is set to True, because the wheelchair is employed as an omnidirection chassis). As previously introduced, during navigation, the target is tracked and consequently, the target position and orientation are updated. The updated position is also transferred to the navigation system to update the planning path to improve the accuracy and allow the system to accommodate dynamic targets. The robotic arm control module is realized using the ROS package provided by the manufacturer. This package has an integrated motion planning algorithm that allows the user to control the robotic arm by simply specifying the position and pose of the end-effector in the robotic arm coordinate system (XYZ coordinate system). To ensure the robotic arm accurately executes operations, we have defined a workspace for the robotic arm (− 200 mm to 0 mm range in the x-axis, 0 mm to 350 mm range in y-axis, and − 150 mm to 300 mm range in z-axis, it is a simple definition that is not represent the official data). The z-value of the target is first checked to roughly confirm the executable of the operation, adjustment in the xy-direction is then made by moving the wheelchair to make the target enter the workspace and then finally the corresponding operation is executed (in cases where the target is not accessible after the wheelchair adjustment, this operation will be rejected by the system).

### Brain–computer interface system

In this study, the users control the mobile platform through the BCI system in three steps: confirming one control mode (autocontrol mode or command control mode), selecting one target or command, and validating the command. As discussed, we have built a smart mobile platform that can recognize the target in the environment and can autonomously complete tasks. This means the user can drive the system by merely selecting one detected target, since the machine will automatically plan a proper solution and complete the task. That is, the autocontrol mode. However, we believe that this kind of mode cannot continuously function, due to situations of the following two main types:

- There is no target within the visual range of the camera, or the system failed to detect and interpret all the targets in the environment.
- There is no target of the user’s desired class in the current scene.

Therefore, to ensure the system is functioning under any conditions, we added the command control mode, which allows the user to control the system directly (there are six commands to control the wheelchair go forward/backwards, translate left/right and rotate left/right) when the autocontrol mode is not available or cannot satisfy the user’s need. To enable the user to confirm their selection, we have also provided a validation method.

#### Working flow

**Fig. 3:**
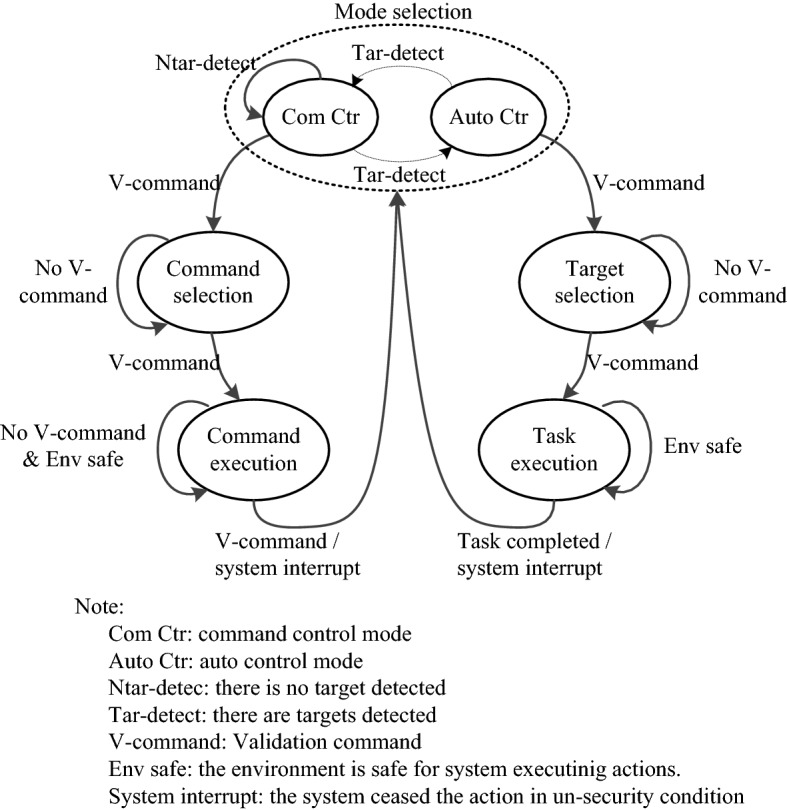
State machine diagram of our system

A state machine diagram is presented in Fig. 3 to illustrate the mechanism by which users can manipulate this system with the autocontrol and command control modes. The system begins at mode selection, and the two modes are alternately preactivated. For each preactivated mode, the user is allocated 3 s to issue a validation command to select the mode. To improve the efficiency of the system, as presented in the diagram, the system will be maintained in the preactivated command control mode if there is no target detected, since target detection is the foundation of the autocontrol mode. As soon as a control mode is confirmed, the targets or predefined commands are displayed through an oddball stimulation interface to allow the user to issue a selection through the P300 BCI. Once the desired command is correctly predicted by the P300 BCI, the user can issue a validation command to confirm his selection, and the system will execute the associated actions. For the autocontrol mode, the actions are ceased after the task is completed, while for the command control mode, the command execution is ceased by another validation command. After the command/task is completed, the system is reset to the selection state mode. Specifically, the system will continuously monitor the system’s commands and status. Any command that causes the mobile system to collide with the environment, or causes a system crash (for example the navigation system enters a deadloop that cannot find a path to the destination), will stop the system and reset the system to its initial state.

#### Graphical user interface

**Fig. 4:**
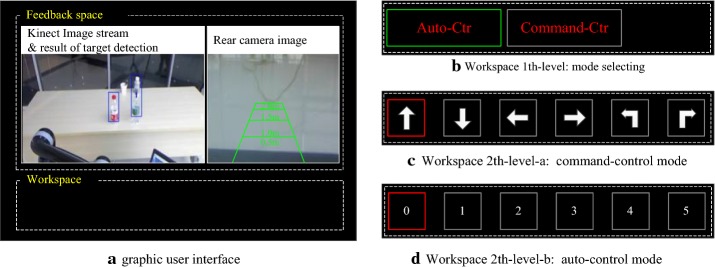
Graphical user interface of our system

A GUI was designed to allow the user to interact with the system. As presented in Fig. 4a, the GUI consists of a feedback space and a workspace. The feedback space displays the information from the environment and the necessary information from the smart system. The right side of the feedback space displays the image stream of the rear camera, which is mounted on the backrest of the wheelchair and points backward. This camera is used to provide the user with backward information during the use of the command control mode. Although the wheelchair is equipped with a navigation system, if the user issues a command to move the wheelchair backward, the rear image is displayed to provide him with visual feedback. The left side of the feedback space displays the image stream of the Kinect camera and the results of the target detection. The detected target is indicated by a blue rectangular box, and the class and the coding number are indicated at the top of this rectangular box. Once the system enters autocontrol mode, the updating image stream will be paused to enable the user to select one target through the BCI system. As long as a target is confirmed by the user, the target is tracked and outlined with a yellow rectangle. The bottom area of the GUI is the workspace for the user to interact with the system. This area of the GUI is a two-level workspace. The first level (see Fig. 4b) shows two alternating lit rectangular boxes that represent “AutoCtr” mode and “CommandCtr” mode. The user confirms the control mode by outputting the validation command when the corresponding rectangular box is lit. After the control mode is confirmed, the second level workspace presents an oddball stimulation interface in which six rectangular boxes are intensified at random. In autocontrol mode (see Fig. 4c), the six rectangular boxes are indicated with number 05, and they are mapped to the targets displayed in the feedback space. During the command driven mode (see Fig. 4c), the six rectangular boxes are presented with arrow graphics that point to left, right, turn left, turn right, forward and backward, which represent the corresponding operations of the wheelchair. Once the second level workspace is activated, the P300 BCI system immediately begins to function, and the target/command predicted by the P300 classifier is indicated by a red rectangular box on the corresponding stimulus. The user confirms the command by issuing a validation command. Once the target/command is successfully selected, the workspace will stop updating, and thus the user knows that the command is accepted by the system. After the task/command is completed, the workspace resets to the first level for mode selection.

#### Data acquisition

**Fig. 5:**
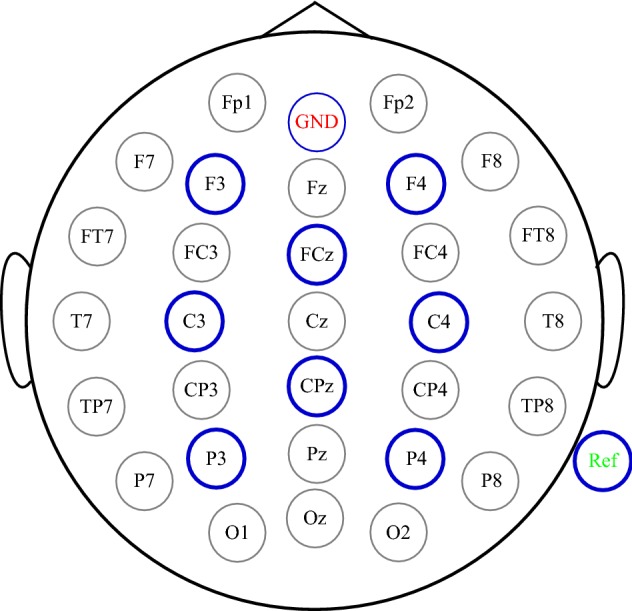
The names and distribution of electrodes. Eight electrodes (bue colour) are employed in our experiment

In this step, EEG signals are recorded using an Actichamp amplifier (Brain product Inc., Germany) through 8 electrodes attached to an EEG cap. The F3, F4, FC1, FC2, C3, Cz, C4, CP1, CP2, P3, Pz and P4 electrodes are included, and the P8 and FPz electrodes are used as the reference and ground, respectively, as illustrated in Fig. 5. The signals are digitized at 500 Hz, while the impedance is maintained below 5 kΩ.

#### P300 BCI

In this study, the P300 BCI is employed to estimate the target attended to by the user. As discussed, six visual stimuli are included in the second level workspace of the GUI. During target/command selection, the six stimuli are randomly intensified for 120 ms with 80 ms intervals. The continuous EEG signals are simultaneously acquired and bandpass filtered between 2 and 25 Hz. After each stimulus onset, the proceeding 800 ms long signal is extracted as an epoch. The stepwise linear discriminant analysis (SWLDA) method is employed to analyse the P300 target signals and nontarget signals. This analysis process is a stepwise regression method that is used to filter the variables that correspond to significant differences between two datasets and provides a weight for each filtered variable. After obtaining the weight vector, each epoch is multiplied by the weight vector to yield a score. This score represents the possibility of a P300 potential being elicited by the associated stimulus. In the command control mode, the target with the highest score in one trial is selected as the output of the classifier. In the autocontrol mode, since there may be less than six detected targets (there are six stimuli in the GUI), only the scores associated with these targets are included in the classification; therefore, the accuracy is improved.

#### Validation command

As introduced in the working mechanism section, a validation command is used to confirm the user’s selections. Therefore, the command should be reliable so that the system can correctly function in practical environments. Based on this consideration, electromyography (EMG) is employed as the signal source to implement this validation command due to the higher signal:noise ratio of this source. The validation command is produced by the user voluntarily clenching his jaw. In [28], the researchers designed a method to classify 5 clenching tasks based on EMG signals extracted from EEG recordings. They evaluated the power spectral density while the users clenched their jaws. The result indicated that signals with a power density between 57 and 77 Hz increased following the clenching action. Thus, according to this research, our method also considers this frequency spectral range. Because only two states need to be recognized, the signal from one channel, i.e., the FCz channel, is acquired. The ongoing signal is extracted within a 200 ms time window and is then bandpass filtered between 55 and 77 Hz. The variance of the signal segment is calculated as the power measurement. A threshold is set to 1500 to identify whether the user is clenching his jaw. The value of this measurement in normal situations is maintained below 100, whereas the value quickly increases to exceed thousands after the user clenches his jaw. To avoid signal fluctuations, a four length first in first out (FIFO) queue is used to accept the latest classifier output, and if the value in the queue is [1,1,0,0], the validation command is confirmed. Therefore, the validation command will be activated after the clenching action is ceased in 0.4 s.

## Experiment

### Participants and preparation

Seven subjects participated in the experiment. Three of these subjects (s1–s3) were patients who were recruited from the Department of Rehabilitation Medicine of the First Affiliated Hospital of Xi’An JiaoTong University in China, and the other four (s4–s7) were healthy people who were recruited from the community and our research unit. s1 is aged 35 years and had a cerebral infarction; he has normal physical function but has poor memory and understanding ability and becomes easily distracted. s2 is aged 32 years and has a spinal injury from a traffic accident that occurred 5 years ago. He has complete upper limb function but no ability to control his lower limbs. s3 is aged 55 years and suffered from a stroke; he therefore has difficulty walking. The other four healthy participants were aged 25–30 years. Among these seven volunteers, the two participants who were recruited from our laboratory had experience using a BCI, and the others had never used a BCI system. This experiment applied for ethics approval to the ethics committee of the First Affiliated Hospital of Xi’an Jiaotong University, and the ethics committee considered that this experiment does not involve an ethics issue. All participants provided written informed consent after the purpose of the study and the task required were explained in detail.

Prior to the online evaluations, the subjects first attended a short BCI training session to calibrate the BCI system. This training consisted of five sets of P300 experiments with each set including 12 trials. The training required approximately 12 min. After the training, the online evaluation experiment was initiated. This experiment required approximately 35 min for one test. To fully test the system, the participants were asked to repeat the test 10 times. Adequate rest was allowed between the two tests. Generally, we ensured that the actual accumulated online time did not exceed 1.5 h. If the user could not complete all the tests in one experiment, the remaining tests were completed on another date.

### Experimental task

**Fig. 6:**
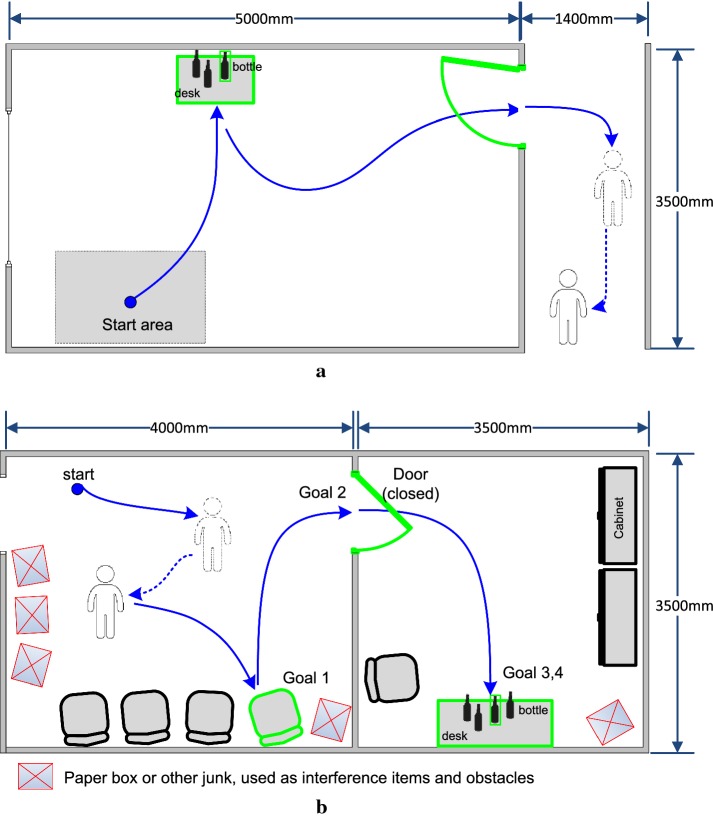
The Experimental environment. **a** Scenario A, in a rehabilitation hospital. **b** Scenario B in our laboratory

The experimental environment simulated a daily domestic environment. The test environments for s1–s3 and s4–s7 were slightly different and are illustrated in Fig. 6. Scenario A (see Fig. 6a) was performed in a sickroom of a hospital; the test environment included a sickroom and a long gallery. In the sickroom, there was a desk with several bottles of water on top. From the sickroom to the gallery, there was an opened door. The experimental task included drinking water and going out of the sickroom to have a conversation with another person. To normalize the experiment, the steps of the experiment were predesigned, and these steps are listed in Table 1. The wheelchair was initialized pointing towards the desk, and the user was instructed to drive the wheelchair to the desk, pick up the bottle (the target bottle was randomly assigned by the experiment assistant after the subject completed the previous task) and drink the water. The user was then required to reverse the wheelchair, turn right towards the door, and pass through the door. After exiting the room, the user was asked to turn right and approach the other person. This other person initially stands still. If the subject initiated the approach of the person with the wheelchair, the person was asked to walk to the end of the gallery. Finally, the subject reached the person to have a 1-min conversation with him.

**Table 1:** The online tasks in scenario A

Steps	Mode	Task
1	Auto-control	Go to the desk
2	Auto-control	Pick up the water
3	–	Drink water
4	Command-control	Reverse
5	Command-control	Turn
6	Auto-control	Pass through the door
7	Command-control	Turn
8	Auto-control	Go to the person (dynamic)
9	–	Have a conversation with the person

Test scenario B (see Fig. 6b) for the healthy subjects was similar to scenario A, but the environment was slightly more complicated to simulate a real-life environment. The rooms were equipped with a few pieces of furniture that included several chairs, a desk, a cabinet and other paper boxes. The steps in the test are listed in Table 2.

**Table 2:** The online task in scenario B

Steps	Mode	Task
1	Auto-control	Go to the person (dynamic)
2	–	Have a conversation with the person
3	Auto-control	Go to the chair
4	Command-control	Turn
5	Auto-control	Pass through the door
6	Command-control	Turn
7	Auto-control	Go to the desk
8	Command-control	Pick up the bottle
9	–	Drink water

### Performance evaluation

To evaluate the system performance, we reference the methods of related studies [3, 21]. The metrics used in this study are as follows:

1. Deductions. Except for false actions induced by the BCI system, each false action accrued one deduction. The deductions are divided into the following two categories:
  Environment perception error (EP): failure to recognize all the targets in the scene; failure to localize and track the target.
  Navigation error (NV): path planning failure (the system was unable to plan a path, although an available path existed); collision with anything during navigation.
  Note: False or inaccurate operation of the robotic arm was considered an inaccurate environment perception.
2. Trials for selecting a command through the P300 BCI (TrP3): the number of trials required by the user to correctly select the assigned target.
3. Time spent to select one command through the P300 BCI (TiP3): the time spent to complete one trial multiplied by the number of trials.
4. Validation time (VT): the time spent from when the desired target command is selected to when the command is validated.
5. False validation (FV): the number of false validations except for the predefined necessary validations.
6. Success rate (SR): the rate of successfully completed tests.

## Results

The metrics of the experiment results were calculated. The seven subjects completed all tests and completed all tasks in each test (the success rate was 100%). During the test, the users’ familiarity with the system quickly improved, and the users quickly became experts at the use of the system and presented good results.

To evaluate the effectiveness of the smart system, the deductions were recorded. As presented in Table 3, all deductions were due to environment perception errors. The subjects averaged 1.14 deductions in the test. As the total test number is ten, the system produced approximately 0.1 environment perception errors in each test. According to the experimental log, the main cause of the deductions was incomplete target detection of the bottles when the wheelchair was far away from the desk. When the mobile system arrived at the desk, the deductions were no longer triggered. We deduced that this source of error may have been caused by the low resolution of the Kinect images and the fact that the object corresponded to too few pixels in the image (recognizing small objects is a common problem for object detection algorithms). Another error that occurred twice was due to the “guest” moving fast while the mobile system moved slowly, consequently, the “guest” moved outside the field of view of the camera, which caused target tracking failure. Other than environment perception deductions, there were no deductions for the whole system, which means that the navigation system worked effectively with a high reliability. The navigation system benefits from the mature algorithms of the navigation package in the ROS. However, some tasks are difficult for the navigation system; for example, the width of the door is 80 cm, while the width of the mobile system is 70 cm. To pass through the door, the system needs an accurately constructed map and fine movement control for the mobile system. The system also benefits from the omnidirectional chassis system, because the chassis allows the path planning system to work in a simple manner. Additionally, the robotic arm system also works accurately and reliably, and thus, the smart system performs well.

**Table 3:** Results of deductions and success rate

Subjects	Deductions		SR (%)
	EP	NV	
S1	0	0	100
S2	1	0	100
S3	0	0	100
S4	2	0	100
S5	1	0	100
S6	2	0	100
S7	2	0	100
Average	1.14	0	100

Tables 4 and 5 list the results of the BCI-related evaluations in scenarios A and B, respectively. To select a target (command), the three patients required an average of 2.04 trials to output the correct command with an average of 7.37 s, as each trial took 3.6 s. Compared with the patients, the four healthy subjects required an average of 1.68 trials to make one correct selection. According to the data from each subject, the subjects commonly required less than two trials to accurately confirm the target through the P300 BCI. Because the total number of stimuli was only six, the subjects could easily maintain their concentration during the short stimulation periods. Subjects one and three required more than two trials to issue a correct command, which was possibly due to their relatively weak ability to concentrate because we observed that they (one has a brain disorder and the other is elderly) had petty actions and distractions during the experiment. Nonetheless, the difference between the patients and healthy subjects was not significant, and they presented similar performances.

**Table 4:** The results of the BCI system evaluation of the three patient subjects

Subjects	TrP3	TiP3	VT	FV
S1	2.05 ± 0.83	7.38 ± 2.98	4.02 ± 2.34	0
S2	1.73 ± 0.71	6.23 ± 2.56	3.15 ± 1.53	0
S3	2.36 ± 0.93	8.50 ± 3.35	4.36 ± 2.11	0
Average	2.04 ± 0.84	7.37 ± 3.02	3.33 ± 2.01	0

**Table 5:** The results of the BCI system evaluation of the three patient subjects

Subjects	TrP3	TiP3 (s)	VT	FV
S4	1.57 ± 0.67	5.65 ± 2.41	2.61 ± 1.09	0
S5	1.95 ± 0.94	7.02 ± 3.38	2.71 ± 2.51	0
S6	1.58 ± 0.75	5.69 ± 2.70	2.10 ± 1.27	0
S7	1.63 ± 0.67	5.87 ± 2.41	1.93 ± 2.02	0
Average	1.68 ± 0.79	6.04 ± 2.84	2.33 ± 2.13	0

For the validation command, the patients and healthy subjects required approximately 3 and 2.33 s to specify the command with standard errors of 2.01 and 2.13, respectively. None of the participants committed any errors in the validation command. Indeed, as introduced in the Methods section, the validation command should be quite reliable, because it is realized through EMG signals. In the test, the drinking and talking tasks were specifically included to test the feasibility of the use of jaw clenching in practical applications. The results revealed that normal actions such as drinking and talking did not induce false detections for the validation command. Regarding the response time of the validation command, we could theoretically detect a validation command within 1 s. However, the results were significantly longer than 1 s. In addition to the time spent, it required more time for the users to confirm the command after seeing the predicted target and then making a decision to execute the clenching action. In other words, the reaction time spent comprised an important part of the total time spent. The results from s1 and s3 fit this theory, since these patients required the longest times to issue the validation command due to their relatively weak reaction abilities. Another phenomenon is that the standard deviation of the subjects’ validation times was large (close to the mean value), which we believe may have been induced by random reactions of the subjects during the experiment.

## Discussion

In this paper, we propose a brain-actuated smart rehabilitation wheelchair that integrates automation and artificial intelligence technology to provide users with an easy-to-use and efficient solution for applications in daily life. As outlined in the Introduction section, several related systems have already been proposed. Table 6, compares our work with work by others. In this table, we have compared our system with those of others based on four factors, including mobility, functionality, dynamics and straightforward use. We used the star symbol ‘*’ to indicate that a system performs well for the corresponding index. The short line symbol ‘–’ indicates relatively weak performance for the corresponding indicator.

**Table 6:** A comparison of our work with related work

Works	Mobility	Capability	Dynamics	Ease-of-use	BCI performance
Rebsamen et al. [20]	–	–	–	*	*
Zhang et al. [21]	–	–	–	*	*
Lopes et al. [22]	–	–	–	*	*
Iturrate et al. [3]	–	–	*	–	*
Our work	*	*	*	*	*

### Mobility

On one hand, mobility means the ability of the wheelchair to flexibly move in a complicated environment. The previous studies are based on the traditional wheel structure; however, for this type of wheelchair, the position adjustment of moving direction is only available along the wheel direction. To improve the flexibility of the system, the omnidirectional chassis was introduced as the base of the wheelchair. It ensures efficient operation in minor position adjustments, especially position adjustments not along the wheel direction. For example, in our system, passing through a door and picking up a bottle commonly require accurate position adjustment, and since the omnidirectional chassis is equipped, the position adjustment is straightforward. For traditional wheel structure-based wheelchairs, minor position adjustments not in the wheel direction are time consuming and may exhaust the user. On the other hand, mobility also means the reachable range of the wheelchair. We have stated that the pure wheelchair system can only move in a free or an enclosed space, however, the potential users of the BCI system are severely disabled people. This means that navigating to a broader space requires the help of others. In our system, a lightweight robotic arm is introduced into the system, and due to its capability to open doors or operate elevator buttons, the reachable range of the system is extended.

### Capability

With the introduction of the robotic arm, our system became a human-like structure with analogue legs and hands. Thus, with proper joint control of these two components, the capability of the system is much enriched. In fact, as we have stated that a specific purpose is commonly associated with the navigation task, our system provides users a complete mobile solution, as our system is capable of dealing with navigation-subsequent tasks.

### Dynamics

Compared to systems relying on pre-determined goals [20–22] in the environment, our system is based on object detection technology that interprets the environment without relying on special environments. Therefore, our system is capable of accommodating different environments. The tests in this study were performed in two different places, and in each test environment, the objects in the environment were randomly placed without special consideration. The results revealed that this system works normally and effectively. In addition, our system is also able to operate with dynamic objects, because image tracking technology is employed. During the test, attending to the walking “guest” is to test the ability of the system to cope with dynamic objects. The test results indicated that the system is able to track a low speed moving object, although tracking performance is limited by the camera’s resolution and the wheelchair’s velocity (objects moving too fast easily exceed the view-sight of the camera, which causes tracking failure). In fact, the system is not a truly dynamic one; during the target selection, the index number of the targets should not vary, because the target selection relies on the mapping relationship between the stimuli’s index and the target’s index. Keeping the index number of targets constant may rely on multiple object image tracking technology, however this is another major concept in the computer vision domain. Additionally, even though Iturrate et al.’s system [3] can work in different environments, it still cannot deal with dynamic objects since the ‘goal’ (defined by a set of grids) of their system is virtual destination without actual meaning.

### Ease-of-use

In Iturrate et al’s. [3] system, they view all the detected objects (using a planar laser scanner) as obstacles to be eliminated in the map; however, we think this approach is not in high accordance with real conditions. The goals of their system are defined by a polar grid. To achieve one target usually requires multiple steps. In contrast to their work, we and Rebsamen [20], Zhang [21], and Lopes [22] employed the target-driven idea that to navigate the wheelchair to the destination, the user only needs to choose a desired goal or select a direction closest to the goal. Thus, the usage of the system is intuitive and user friendly. Beyond navigating somebody from place A to place B, our system tries to understand the real intent of the user by considering the attributes of the target, the user’s behaviour and state, and the context. In other words, our system can intelligently provide a proper solution that can satisfy the user’s requirement. In addition, our system is further able to predict the most likely selected target by the user and therefore further improve the efficiency of the system. We designed rules to assign a priority to each object; for example, a person has a higher priority than a chair, and closer objects are assigned higher priorities. The objects are sorted by priority, and the objects sorted lower than sixth are ignored (for this reason, we defined only six items in the P300 BCI). The object with highest priority is selected by default. Thus, if the default object fits the user’s intent, the user can directly issue a validation command without engaging in the selection process. However, to fully test the system, this rule was not applied during the test. In summary, with our system, the user needs only to perform a few commands to achieve his goal, and the system is friendly and efficient.

### BCI performance

As in the other cited works, we also employed the P300-based BCI to confirm the subject’s selection. There are no significant differences in BCI performance between our systems. Actually, previous studies [29] have already demonstrated that most people can achieve high accuracy after a short training duration. In addition, in our experiment, the two brain injured patients also did not present significant differences in BCI use compared to healthy subjects. In addition to the P300 BCI, EMG was used in the system to validate the commands. The signal-to-noise ratio of EMG signals is much higher than that of EEG signals, and therefore, using EMG signals in the system to validate the command is a good choice. In our system, the jaw clenching action is employed to export the validation command, and the results indicated that this signal is reliable and fast. Because this signal is activated by a jaw clenching action, swallowing and talking were tested, and the results indicated that these actions did not induce false detections. During more in-depth testing, only eating caused false detections. Therefore, the system is limited in that it cannot be used while eating. Fortunately, the use of a mobility system while eating is not advocated. Indeed, this problem can be solved by adding a “switch” to turn off/on the system with a special clenching pattern.

*In addition to the five indicators*, there is another possible advantage of our system, as we employed ROS to construct the programme. The ROS provides a standard methodology and message format for communication between modules. Each node is an independent process that does not rely on a special programming environment. Therefore, it is easy and convenient to upgrade and extend the system, which is an important property for the system’s extension and application.

### This system also has certain limitations

1. We stated that our system is a dynamic system that can accommodate different environments, because we have employed a deep-learning-based target recognition (YOLO) algorithm for real time recognition of objects in the environment. To make sure the algorithm can truly accommodate different situations, the algorithm mode should be well trained, however this would rely on a large-scale database. Obviously, such a database is rather expensive and time consuming to build. Fortunately, there are some open source image databases, such as COCO and ImageNet [30]. These databases provide images of many types of objects in various environments. The model can be trained using these databases and can even be simultaneously trained across multiple databases. The YOLO9000 is a good example; the model was simultaneously trained on the COCO and ImageNet databases and therefore achieved recognition of 9000 objects.
2. Another limitation is that the autocontrol mode is limited by the visual sight of the camera, since the target detection is based on the image of the Kinect; therefore, we have to provide an added command control mode to drive the system when the auto control mode is not available. Of course, this is also a common problem of the system by Iturrate et al. [3] as we are only concerned with the current information regarding limited visual sight. Fortunately, to solve this problem, there are several methods. The first method is to equip four Kinects in four directions to provide information on the surrounding environment. The second method would be to employ the idea of simultaneous localization and mapping (SLAM) technology to reconstruct a global map based on each measurement frame. Thus, the system can provide the user a global map that contains not only the scenario of current visual sight but also other targets out of the visual sight.

## Conclusion

This article presents a brain-actuated smart wheelchair system. The system is intelligent and provides efficient and considerate services for users. To test the system, three patients and four healthy subjects were recruited to participate in a test. The results prove that the system works smartly and efficiently; with this system, users only need to issue small commands to get considerate services. This system is of significance for accelerating the application of BCIs in the practical environment, especially for patients who will use it for rehabilitation applications.

## Abbreviations

EEG: electroencephalogram
BCIs: brain–computer interfaces
SSVEP: steady state visual evoked potentials
MI: motor imagery
ROS: robot operating system
FPS: frame per second
GPU: graphic processing unit
COCO: Common Objects in Context dataset
PCA: principal component analysis
KCF: kernelized correlation filter
GUI: graphical user interface
SWLDA: stepwise linear discriminant analysis
EMG: electromyography
FIFO: first-in-first-out
